# Changes in dietary habits and BMI *z*-score after a 6-month non-randomized cluster-controlled trial among 6–12 years old overweight and obese Norwegian children

**DOI:** 10.29219/fnr.v67.9617

**Published:** 2023-12-12

**Authors:** Tonje Holte Stea, Mario Vianna Vettore, Bente Øvrebø, Eirik Abildsnes

**Affiliations:** 1 Department of Health and Nursing Science, University of Agder, Kristiansand, Norway; 2 Department of Health and Inequalities, Norwegian Institute of Public Health, Oslo, Norway; 3 Department of Psychosocial Health, University of Agder, Grimstad, Norway

**Keywords:** overweight, obesity, children, family involvement, dietary intervention

## Abstract

**Background:**

Effective prevention programs to address the high prevalence of childhood overweight and obesity and the concomitant health consequences have been warranted.

**Objective:**

To improve dietary habits and weight status among Norwegian children with overweight/obesity in the primary care setting.

**Design:**

A 6-month non-randomized cluster-controlled intervention among 137 children, aged 6–12 years, with overweight/obesity and their parents. Intervention and control groups were recruited by public health nurses and followed-up by 12 Healthy Life Centers across Norway. The intervention group received individual family counseling and participated in nutrition courses and physical activity groups. A frequency questionnaire assessing sociodemographic characteristics and dietary habits was completed by the parents. Trained public health nurses measured height and weight using standardized methods to calculate body mass index (BMI) and BMI *z*-scores.

**Results:**

The intervention resulted in an increased odds of consuming evening meals (OR: 3.42), a decreased availability of salty snacks (β = –0.17), a decreased intake of salty snacks (−0.18), an increased consumption of water (β = 0.20), and a decreased estimated total intake of energy (β = –0.17), carbohydrates (β = –0.17), mono- and disaccharides (β = –0.21), sucrose (β = –0.24), and saturated fatty acids (β = –0.17). The intervention directly predicted lower BMI *z*-score (β = –0.17), and post-treatment levels of energy (β = –0.65), saturated fat (β = 0.43), and total carbohydrates (β = 0.41) were directly linked to BMI *z*-score after intervention. Age and sex were indirectly associated with BMI after intervention through energy and saturated fat intake.

**Conclusions:**

The intervention had a beneficial impact on nutrient intake and weight status among children with overweight/obesity. These findings provide support for implementing complex intervention programs tailored to local primary care settings.

**Trial registration:**

Clinicaltrials.gov, NCT02290171. Registered 13. November 2014, https://clinicaltrials.gov/ct2/show/NCT02290171

## Popular scientific summary

- A 6-month family-based intervention study was tailored to improve lifestyle habits among overweight and obese children in primary health settings.
- The intervention resulted in improved eating behaviors and reduced energy intake.
- A decrease in BMI z-score was observed in the intervention group compared to the control group after the intervention period.
- This study provides support for implementing complex intervention programs tailored to local primary care settings.

During the past decades, the prevalence of childhood overweight and obesity has increased worldwide (1), but evidence suggests a stabilization in the prevalence of overweight and obesity in developed countries (2). Stabilization of overweight and obesity prevalence is also supported by results from a Norwegian longitudinal study among 8- to 13-year-olds, which reported that 16% of the adolescents were overweight and 3% were obese, and that the prevalence of overweight and obesity in adolescence seemed to be established during childhood (3). Childhood overweight and obesity often persists into adulthood and may lead to an increased risk of developing chronic disorders during early adulthood (4,5,6). Childhood obesity should be classified as a chronic disease in itself according to the Childhood Obesity Task Force (COTF) of the European Association for the Study of Obesity (EASO) (7). Such approach aims to increase the individual and societal awareness related to childhood obesity and improve early diagnosis and intervention (7). Nevertheless, type 2 diabetes, hypertension, dyslipidemia, metabolic syndrome, and obstructive sleep apnea are adverse health outcomes associated with overweight in children and adolescents (8), and the aforementioned risk factors might result in premature heart disease, cancer, and adult diabetes (6).

The link between overweight/obesity and poor health among children is complex due to the interrelationships among genetic, biological, behavioral, social, cultural, and environmental influences (9, 10). The main postulated explanations for the childhood obesity epidemic are unfavorable dietary habits, increased sedentary behavior, and reduced physical activity, and the impact of these risk behaviors on weight status is modified by age and sex (11). Findings from a study among Norwegian 11-year-olds indicated sex differences in behavioral correlates of weight status, but not for weight status itself (12). In a sample of Australian children, results also indicated changes in environmental factors with age, and that family food environment among older children was associated with children’s body mass index (BMI) *z*-score and weight status cross-sectionally and longitudinally (13).

In addition, social inequalities related to body composition are well established, with greater fat mass, lower fat-free mass, and higher prevalence of overweight and obesity among disadvantaged children (14, 15). Studies have confirmed that diet quality may explain this relationship as children in the higher socioeconomic categories consume a healthier diet (16, 17) and have a healthier body weight (18). A study among Norwegian children has also confirmed socioeconomic inequalities in children’s weight, height, and BMI trajectories (19). Moreover, findings from a study among children aged 6–10 years in the US suggested that the increase in food responsiveness and emotional overeating in obese children was influenced by maternal education (20).

A recent review has reported that first-line treatment approaches to tackle childhood obesity include family-based behavioral interventions focusing on improving healthy lifestyle habits, including dietary habits, physical activity, sedentary behaviors, and sleep quality, underpinned by behavior change strategies (21). In Norway, measurements of height and weight among all children entering 1st, 3rd, and 8th grade are routinely collected every year at school by public health nurses. According to the national health authorities, public health nurses are obliged to measure and report childhood overweight and obesity to parents and provide further advice on weight management (22). However, national guidelines do not provide specific recommendations or practical guidelines for the prevention and management of overweight and obesity in children (23).

A previous systematic review has emphasized that in general, national screening programs aiming to identify children with overweight/obesity are not justified without follow-up effective intervention programs (24). However, findings from most of the published dietary intervention studies aiming to prevent or reduce childhood obesity have shown no or limited effects on weight reductions (25). On the other hand, another systematic review concluded that complex lifestyle interventions targeting children and adolescents with overweight/obesity and involving structured strategies for change in diet and physical activity may reduce BMI and BMI *z*-score compared with less complex interventions (26). A Cochrane review also confirmed that multi-component behavior-changing interventions may be beneficial in achieving small, short-term reductions in BMI and BMI *z*-score in children aged 6–11 years, but the overall quality of the evidence was low or very low partly due to differences in study design, setting, parental involvement, and inconsistent results (27). The ecological model for health promotion has highlighted the importance of the influence of both individual and environmental factors, including parent–child interaction, when planning health promotion interventions for children (28). Although family-based behavioral programs, which specifically target parents as the exclusive mediator of obesity treatment of children, are known to be an effective approach to reduce childhood obesity, few studies have included parents as behavioral agents (29). Due to the lack of high-quality evidence, further research is warranted in which health practitioners can work with parents as agents of change and focus on fostering positive parenting skills in order to support health behaviors in their children (30).

Our research group has previously published results from a systematic and evidence-based pilot study developed to specifically target children with overweight/obesity and their families with program components tailored to the local context in Norwegian municipalities (31). The theoretical model that we have developed hypothesized that the intervention, child’s age, and sex would be directly associated with nutrients and BMI *z*-score after the intervention. Indirect effects of age, sex, and the intervention on BMI *z*-score after intervention mediated by nutrients were also hypothesized. Based on findings from the pilot study, we developed a family-based, multi-component program to improve healthy lifestyle habits among children and adolescents in a primary care setting. The main aim of this present study was to examine possible effects of the developed intervention on dietary habits and weight status among children with overweight and obesity.

## Materials and methods

### Study design and participants

This 6-month non-randomized cluster-controlled trial was based on the results of a 1-year pilot study, which was systematically developed and implemented as a tailored family-based intervention to improve lifestyle habits, by encouraging physical activity and healthy dietary habits, enhancing parental self-efficacy, and enhancing family engagement and parent–child relationships (31). In the present study, we evaluated the possible effects of the 6-month intervention on meal pattern, food and beverage availability and consumption, nutrient intake, and weight status among children with overweight/obesity.

The study population consisted of children with overweight/obesity and their families, recruited from October 2014 to September 2016 by public health nurses after routine screening of weight and height in preschoolers and among 3rd grade pupils. The International Obesity Task Force (IOTF) BMI cut-offs and IOTF LMS parameters were used to categorize BMI and calculate BMI *z*-score, respectively (32). Healthy Life Centers (HLCs) and Public Health Clinics (PHCs) in the municipalities Bergen (two districts), Sola, Sandnes, Stavanger, Søgne, Kristiansand (three districts), Grimstad, Horten, and Oslo (two districts) were responsible for recruiting families and conducting the intervention in collaboration with local sports clubs. The families participating in the present study were recruited based on assessment of overweight and obesity after standard measurement of children’s height and weight during school hours in 1st and 3rd grades. The recruitment process in regular services in several different municipalities did not allow the identification of the total number of children and families invited.

The intervention group and the control group were selected without any random pre-selection processes. HLCs and PHCs with an existing infrastructure facilitating the implementation of the intervention program recruited participants to the intervention group, whereas other HLCs and PHCs recruited participants to the control group. All participants in the control group were informed that they would receive the intervention program by HCLs and PHCs in their local community after serving as a control group for 6 months. Thus, the control group served as waiting list group and did not receive any counseling during the intervention period.

A total of 164 children and adolescents between 6 and 12 years old and their families agreed to participate in this study (control group: *n* = 73, intervention group: *n* = 90). Of them, a total of 77 participants from the intervention group and 66 participants from the control group completed the intervention period. Reasons for loss to follow-up included lack of time, moved away, conflicting time schedules, and participation in other leisure time activities.

Based on results from a previous intervention study targeting obese children for the promotion of healthy lifestyle habits and weight reduction, we conducted sample size calculations (33). The final sample of 143 participants, to detect effect size at least 0.15 (medium effect size) in a multiple regression analysis involving six independent variables with 5% Type I error probability, would lend a power of 94% (34).

Prior to participation in the present study, the children and their parents received oral and written information about the study. Parents signed a written consent form before completing an online questionnaire providing information on their children’s lifestyle behaviors as well as demographic and sociodemographic data (e.g. sex, age, and educational attainment). Results from each participant were registered with a unique identification code provided by the local HCLs, which made it possible to link data collected during the intervention period and ensure anonymity, as the identity of individual participants was not known to the researchers. The Regional Committee for Medical and Health Research Ethics approved the study (no. 2013/1291), which was conducted in accordance with the Helsinki Declaration of 1975, as revised in 2008.

### Deviation from the protocol

Originally, we intended to perform a cluster randomized clinical trial among overweight and obese children between 6 and 10 years old and their parents. However, the present intervention study used a quasi-experimental controlled design, as it was only feasible to recruit participants to the intervention group and the control group without any random pre-selection process. For ethical reasons, we also used a waiting list design, which did not allow long-term controlled follow-up as the control group received the same intervention as the intervention group with a 6-month delay. Finally, we decided to include a wider age range than described in the protocol, as the municipal healthcare institutions included in the present study had focus on screening and treatment of overweight and obesity in children between 6 and 12 years old.

### The intervention program

Results from the systematic and evidence-based approach used for the development of this family-based intervention study have been described in the study protocol (31). We decided to focus on changing three key behaviors, i.e. level of physical activity, diet, and self-regulation skills. Furthermore, these behaviors were translated into 10 main program objectives: 1) increase level of physical activity, 2) decrease sedentary time and screen time viewing, 3) establishing regular meal patterns, 4) increasing number of family meals, 5) regulate/decrease portion sizes, 6) increase intake of healthy food and beverages and decrease intake of unhealthy alternatives, 7) establish adequate duration of sleep, 8) increase self-esteem, 9) strengthen autonomy support (parents), and 10) strengthen autonomous regulation and self-efficacy (parents). In addition, two subsidiary program objectives were described: 1) reducing BMI *z*-score and 2) improve self-perceived health.

Based on experience from the pilot study, we aimed at developing an intervention tailored to individual needs, enhancing parental motivation, and taking environmental factors into account. Thus, an ecological approach was used when developing the intervention, but the Self-Determination Theory (SDT) served as the main theoretical framework (35). According to SDT, different types of intrinsic and extrinsic motivation can underlie one’s behavior. Intrinsic motivation refers to the inherent satisfaction related to the behavior, which was the central component of the motivational interview (MI) to enhance distinct attributes of the participants, such as empowerment and self-efficacy. Extrinsic motivation is defined as engaging in a behavior for instrumental reasons or to obtain some outcome disassociated from the behavior per se. It may include some motivation of personal value, including exercising to maintain good health, such as the physical activity sessions of the intervention. All participants in the intervention group received tailored individual (face-to-face) counseling and follow-up by certified health personnel using MI as a counseling technique in consultations to promote internal motivation, empowerment, and mastery of health (36). During the first meeting, an individual plan (IP) defining a maximum of three main goals for behavioral change followed by several more specific sub goals in the IP for each family during the intervention period was created in collaboration between parents and health personnel. A minimum of three and a maximum of eight individual consultations were offered to support all participating families in reaching these goals during the intervention period. Results from the previously published pilot study indicated that both parents and health personnel responsible for the individual consultations were satisfied with the scope of the IP, the frequency of the individual consultations, and the use of MI as a technique designed to change specific health behaviors (31).

In addition to individual counseling and follow-up, parents and their children attended four-five face-to-face courses, developed by a nutrition scientist that included both theoretical and practical learnings sessions. All courses were tailored to meet the specific needs and challenges regarding nutritional knowledge, attitude, skills, self-efficacy, and intentions toward improving healthy dietary habits among this target group. Methods and strategies used during individual counseling and group-based courses for the promotion of healthy eating also focused on changing home environment by mobilizing social support, provide positive reinforcement, and focus on the importance of high availability of healthy food choices and low availability of unhealthy food and beverages. In addition, children in the intervention group participated in physical activity sessions on a weekly basis (1–2 times per week), aiming at increasing moderate-to-vigorous intensity physical activity (MVPA) and improving motor control. The physical activity program was developed by physical activity researchers, who were experienced in planning, implementing, and evaluating the effect of physical activity sessions targeting children with overweight and obesity. Detailed information about program components and materials has been described in detail elsewhere (31). Intervention adherence and fidelity according to the protocol were controlled and approved for all participants by health personnel responsible for the individual consultations and group-based courses, and certified activity leaders responsible for physical activity groups.

### Data collection

Weight and height were measured at baseline and after the 6-month intervention period by trained health professionals at the participating HLCs and PHCs. Information about sociodemographic background, meal pattern, and food and beverage availability and consumption were provided by parents completing online questionnaires using Survey Exact. Each participant was given a personal identification-number used to identify and link participants’ data collected in the study.

### Questionnaire

A self-administered food frequency questionnaire (FFQ) based on a Danish FFQ for children and adolescents was filled out by all parents at baseline and after a 6-month intervention period. The FFQ has been validated prior to this study (37) and consisted of 146 questions on the average consumption of food and beverage items during the past 4 weeks. These food and beverage items were divided into different categories (i.e. beverage and dairy products, bread and cereals, spread/cold cuts, fish and meat, dinner meals, side dishes, fruit and vegetables, dessert, cakes, and snacks).

Information regarding intake of food items, dinner meals, side dishes, and beverages was assessed with the following question: *How often does your child consume the selected food item/dinner meal/side dish/beverage*? For specific food items, each question had seven response options: *never, 1–3 times per month, once a week, 2–3 times per week, 4–6 times per week, and once or more times a day*. Dinner meals and side dishes used the interval ranges: *never, 1–3 per month, 1 per week, 2–4 per week, and more than 4 times per week*. For the consumption of beverages, the interval ranges were *never, 1–3 glasses per month, 1 glass per week, 2–6 glasses per week, 1 glass per day, 2–3 glasses per day, and more than 3 glasses per day.* The respondents reported their consumption of food items, dinner meals, side dishes, and beverages in ‘units per month’, ‘units per week’, or ‘units per day’. The unit measurements differed between items, but most questions were related to a standard portion size (e.g. cup of coffee, a piece of bread, and an apple). For some questions, extra information was provided (e.g. maize = 2 tablespoons or soda = 0.5 L).

When calculating nutrient intake for each participant, the standard portion included in the FFQ (e.g. glass of milk), which was coded according to the Norwegian standards (38) in grams, was multiplied by the intake frequency registered in the FFQ. In addition, each food and beverage item had a food-code linkage corresponding to the numbers in the Norwegian food composition table. For dishes, we adjusted for estimated nutrient loss as a result of using different cooking procedures. Finally, we used FOOD-Calc and the Norwegian food composition table (39, 40) to calculate total nutrient intake for each participant at baseline and 6-month follow-up. In the present study, we reported total energy intake (kJ), total intake of proteins (g), and intake of carbohydrates (g), including total carbohydrate intake (g) and intakes of starch, mono-, and disaccharides (g), sucrose (g), and fiber (g/1,000 MJ). We also reported intake of fat, including total fat intake, and intakes of cholesterol (mg), saturated fatty acids (SFAs) (g), monounsaturated fatty acids (MUFAs) (g), polyunsaturated fatty acids (PUFAs) (g), *n*-3 fatty acids (g), and *n*-6 fatty acids (g). Finally, we present the percentage of total daily energy intake (E%) for protein, total fat, SFAs, MUFAs, PUFAs, total carbohydrate, and sucrose.

Information about the children’s habitual consumption of main meals and family meals was obtained by asking respondents the following question: *How often do you usually eat breakfast, lunch, dinner, and evening and family meals each week?* Each question had five response options: *never or seldom, 1–2 times per week, 3–4 times per week, 5–6 times per week, and daily.* For statistical analyses, those who reported a daily intake of these main meals or having family meals daily were classified as regular breakfast-, lunch-, dinner-, and evening and family meal consumers, whereas those who omitted these main meals and family meal at least once a week were classified as irregular (family) meal consumers.

Information regarding food and beverage availability (availability of candy, salty snacks, diet-soda, sugar-sweetened soda and fruits and vegetables) was assessed using the following question: *How often are the selected food item/beverage available?* Each question had 10 response options: *never, less than once a month, less than once a week, once a week, two times a week … up to seven days a week.* In the statistical analysis, they were scored 0, 0.1, 0.5, 1, 2, 3 … 7, to reflect the number of days per week these food and beverages were available for consumption at home.

The questionnaire also included sociodemographic information, such as child’s age and sex, and parental education. Parental education level was assessed using the following question: *What level of education do you have, answer for yourself and your partner.* Both questions (for yourself and your partner) had six response options: *elementary school <7 years, elementary school 7–10 years, vocational school/high school <3 years, high school – 3 years, 3 years of high school, college or university* ≤*4 years, and colleges or university ≥4 years.* These response alternatives were then trichotomized to reflect the following educational level for both parents: primary school, high school, and college/university. Parental education was included in statistical models as previous systematic reviews have reported an association between low socioeconomic position and higher levels of obesity in high-income countries, especially when parental education was used as a measure of socioeconomic position (41).

### BMI and BMI z-scores

Trained public health nurses were responsible for weight and height measurements using standard calibrated scales and stadiometers to determine height to the nearest 0.1 cm and weight to the nearest 0.1 kg. All children were weighted wearing light clothing and without shoes. BMI was calculated (body mass (kg)/height^2^ (m^2^)), and BMI *z*-scores were assigned to each participant (32).

### Statistical analysis

Difference in prevalence of overweight and obesity and difference in sociodemographic characteristics between the intervention and the control group at baseline were analyzed using the independent sample *t* test for the continuous variable (age) and the Chi-Square Test for categorical variables. In this study, five outcomes were considered to evaluate the impact of the behavior intervention, considering the control group as reference category: 1) meal patterns (categorical variables according to the frequency of daily breakfast, lunch, dinner, and evening and family meals), 2) availability of selected food and beverages (continuous variables based on the number of days per week of sugar-sweetened soda, diet soda, fruit and vegetables, sweets, and salty snacks), 3) consumption of food and beverages (continuous variables (g/day) according to the amount of different food and beverages intake), 4) BMI (continuous variables using kg/m^2^ and *z*-score), and 5) nutrient intake (continuous variables of intake of energy (kJ) and other different measures in g and E%). Differences in frequency of meal consumption between the intervention group and the control group at baseline and after the intervention period were compared using the Chi-Square Test. The effect of the intervention on different meal patterns was tested by using multi-level logistic regression models. Significance of differences between the intervention group and the control group at baseline and after the intervention in mean counts of food and beverage availability and consumption, nutrient intake, BMI, and BMI *z*-score period was analyzed using the independent sample *t*-tests. The effect of the intervention on food and beverage availability, consumption, estimated nutrient intake, BMI, and BMI *z*-score was tested by using multiple multi-level linear regression models. The multi-level structure of analysis including 143 participants (level 1) grouped into the 12 HLCs and PHCs (level 2) was initially tested. A two-level random intercepts and fixed-slopes null model with individuals nested within HLCs and PHCs were fitted using the maximum likelihood method to assess the variance and standard error for BMI *z*-score after intervention across HLCs and PHCs (random effects). The variation of BMI *z*-scores after intervention at HLCs and PHCs level in the null multilevel model was not statistically significant when the variance and standard error for the outcome at HLCs and PHCs level (random effects) were tested (Wald *Z* test = 0.971, *P* = 0.332). Therefore, multi-level analysis accounting for HLCs and PHCs level was not used. All models were adjusted for baseline scores, age, sex, and maternal and paternal education.

**Fig. 1:**
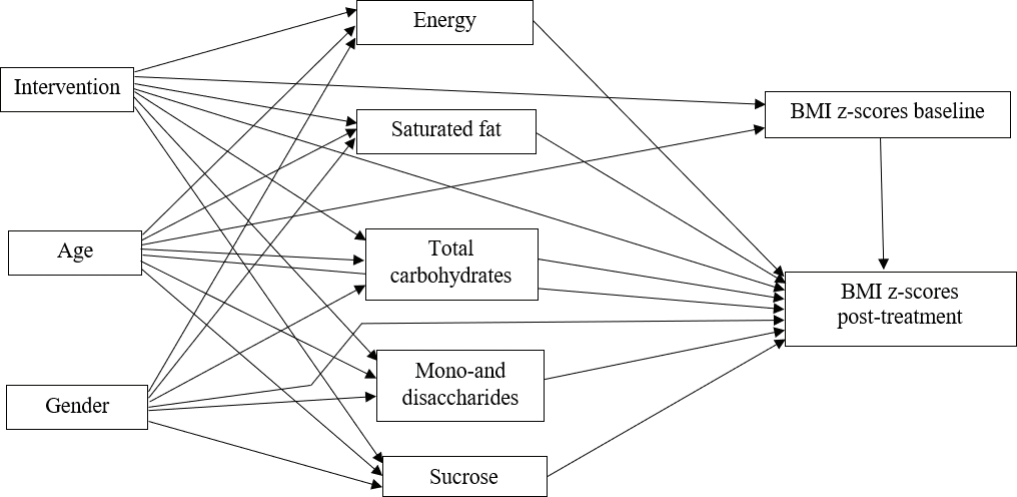
Full theoretical model on the relationships between sex, age, intervention, nutrients, and BMI *z*-score. BMI: body mass index.

Path analysis was used to test the direct and indirect relationships between the intervention, child’s age and sex, nutrients, BMI *z*-score at baseline, and BMI *z*-score post-treatment, according to the theoretical model (Fig. 1). The standardized direct effects refer to a direct path from one variable to another, whereas indirect effects are detected when one variable was the pathway between two other variables. The theoretical model hypothesized that the intervention and child’s age and sex would be directly associated with nutrients and BMI *z*-score after the intervention. Indirect effects of age, sex, and the intervention on BMI *z*-score after intervention mediated by nutrients were also hypothesized. Initially, the full model using the maximum likelihood method was tested for identification. Then, non-substantial direct paths between variables (*P*-value > 0.10) were removed, and the parsimonious model was re-estimated to obtain standardized effects, standard errors, and *P*-values. The overall model fit of the full and parsimonious models was considered adequate according to the Comparative Fit Index (CFI) ≥ 0.90 (42). Statistical analyses were performed using the IBM SPSS version 25 and Stata program version 17 (StataCorp LP, College Station, TX, USA). For all analysis, *P*-value <0.05 was considered as statistically significant.

## Results

### Participant characteristics

According to results presented in Table 1, no differences in age, sex, and maternal education were shown at baseline between the intervention and the control groups. The paternal educational level was somewhat higher among participants in the intervention group compared to the control group (*P* = 0.041).

**Table 1:** Sociodemographic characteristics of the intervention and control group

Sociodemographic characteristics	Intervention group (*n* = 77)	Control group (*n* = 66)	*P* *
Age (years)	8.6 (8.1, 9.0)	8.1 (7.8, 8.4)	0.067
Sex (girls)	41 (59)	35 (59)	0.081
Maternal educational level			
Primary scool	6 (8.7)	3 (5.2)	0.461
High school	31 (44.9)	32 (55.1)	
College/University	32 (46.4)	23 (39.7	
Paternal educational level			
Primary scool	8 (11.6)	9 (16.1)	0.041
High school	30 (43.5)	34 (60.7)	
College/University	31 (44.9)	13 (23.2)	

* Tested using independent sample *t*-test for continous variable (age) and chi-square test for categorical variables.

### Change in meal pattern

Table 2 shows an increased odds of consuming evening meals among participants in the intervention group than those in the control group (OR = 3.42 (95% CI: 1.21, 9.66)). No significant changes in consumption of breakfast, lunch, dinner, and frequency of family meals were reported as a result of the intervention.

**Table 2:** Changes in meal pattern during the 6-months intervention period

Daily meals	Baseline			6 months of follow-up			Within-group proportion changes after 6 months			
	Intervention group	Control group	*P* *	Intervention group	Control group	*P* *	Intervention group	Control group	*OR* (95% CI)	*P* ^#^
Breakfast, *n* (%)	49 (71)	44 (75)	0.652	54 (78)	42 (71)	0.357	5 (7)	−2 (−4)	2.45 (0.79, 7.58)	0.120
Lunch, *n* (%)	52 (75)	44 (75)	0.918	52 (75)	46 (78)	0.729	0 (0)	2 (3)	0.59 (0.21, 1.66)	0.313
Dinner, *n* (%)	56 (81)	51 (86)	0.421	61 (88)	49 (83)	0.385	5 (7)	−2 (3)	1.27 (0.34, 4.79)	0.720
Evening meal, *n* (%)	35 (51)	37 (63)	0.173	47 (68)	31 (53)	0.072	10 (17)	−6 (−10)	**3.42 (1.21, 9.66)**	**0.020**
Family meals, *n* (%)	39 (57)	34 (58)	0.900	42 (61)	35 (59)	0.857	3 (4)	1 (1)	1.29 (0.52, 3.25)	0.580

* Difference in meal pattern between the intervention group and the control group was analyzed using Chi-square tests.

# Intervention effect analyzed as difference in mean scores at baseline and after 6 months of follow-up in each variable (of each group) adjusted for baseline scores, age, sex, maternal- and paternal educational level using logistic regression models.

CI: confidence intervals; OR: odds ratio.

*p*-value < 0.05 was considered as statistically significant.

### Change in food and beverage availability and consumption

Table 3 indicates that the participants in the intervention group significantly decreased the availability of salty snacks than those in the control group (β = –0.18 (95% CI: –1.61, –0.15)). Furthermore, Table 4 shows a significant decrease in the reported daily consumption of salty snacks in the participants in the intervention group than those in the control group (β = –0.18 (95% CI: –7.69, –0.07)). A significant increase in daily water consumption was also reported by the intervention group compared to the control group (β = 0.20 (95% CI: 17.91, 466.42)). We revealed no other significant changes in availability or consumption of different food groups or beverages during the intervention period.

**Table 3:** Changes in availability of selected food and beverages during the 6-month intervention period

Availability of food and beverage	Baseline			6 months of follow-up			Within-group mean changes after 6 months			
	Intervention group	Control group	*P* *	Intervention group	Control group	*P* *	Intervention group	Control group	*β* (95% CI)	*P* ^#^
Sugar-sweetened soda (days/week)	1.3 (0.8, 1.8)	1.6 (1.0, 2.2)	0.183	1.1 (0.6, 1.6)	1.6 (1.0, 2.3)	0.084	−0.2 (−0.7, 0.3)	0.17 (−0.3, 0.6)	−0.08 (−0.99, 0.27)	0.261
Diet soda (days/week)	1.5 (0.9, 2.0)	2.0 (1.2, 2.7)	0.558	1.3 (0.8, 1.7)	1.8 (1.1, 2.4)	0.561	−0.9 (−1.6, −0.2)	−0.9 (−1.6, −0.1)	−0.05 (−0.95, 0.48)	0.516
Fruit and vegetables (days/week)	6.2 (5.7, 6.6)	6.4 (6.0, 6.8)	0.525	6.4 (6.1, 6.8)	6.3 (5.8, 6.8)	0.736	0.0 (−0.2, 0.3)	0.3 (−0.2, 0.7)	0.07 (−0.40, 0.84)	0.476
Sweets (days/week)	2.5 (1.9, 3.1)	2.6 (2.0, 3.2)	0.420	2.1 (1.6, 2.7)	2.7 (2.0, 3.4)	0.314	−0.4 (−1.0, 0.2)	0.3 (−0.3, 0.9)	−0.13 (−1.41, 0.15)	0.110
Salty snacks (days/week)	2.3 (1.7, 2.9)	2.0 (1.4, 2.6)	0.850	1.7 (1.2, 2.2)	2.3 (1.7, 3.0)	0.191	−0.6 (−1.1, −0.1)	0.5 (−0.1, 1.1)	**−0.18 (−1.61, −0.15)**	**0.019**

Data are presented as mean (95% CI).

* Differences in food and beverage availability between the intervention group and the control group, analyzed using independent sample *t*-tests.

# Intervention effect analyzed as difference in mean scores at baseline and after 6 months of follow-up in each variable (of each group) adjusted for baseline scores, age, sex, maternal-, and paternal educational level using linear regression models.

CI: confidence intervals; OR: odds ratio.

*p*-value < 0.05 was considered as statistically significant.

**Table 4:** Changes in consumption of food and beverages during the 6-month intervention period

Intake	Baseline			6 months of follow-up			Within-group mean changes after 6 months			
	Intervention group	Control group	*P* *	Intervention group	Control group	*P* *	Intervention group	Control group	*β* (95% CI)	*P* ^#^
Water (g/d)	612.1 (562.3, 661.8)	554.0 (490.4, 617.5)	0.153	659.5 (623.4, 695.6)	576.7 (524.5, 628.8)	0.010	47.4 (5.3, 89.5)	22.7 (−34.1, 79.5)	**0.20 (17.91, 466.42)**	**0.010**
Sugar-sweetened soda (g/d)	154.0 (121.5, 250.6)	186.1 (121.5, 250.6)	0.439	85.0 (54.8, 115.1)	130.8 (81.5, 180.2)	0.116	−69 (−119.4, 18.7)	−55.3 (−100.1, −10.4)	−0.11 (−82.58, 14.57)	0.168
Diet soda (g/d)	34.2 (19.2, 49.1)	38.4 (20.9, 55.9)	0.713	39.6 (21.9, 57.3)	26.1 (13.3, 39.0)	0.220	5.4 (−7.4, 18.3)	−12.3 (−24.9, 0.3)	0.11 (−2.18, 31.16)	0.088
Fruit and berries (g/d)	150.0 (127.9, 172.8)	156.1 (126.8, 185.3)	0.758	134.7 (118.0, 151.5)	149.8 (119.8, 179.8)	0.383	−15.6 (−35.0, 3.8)	−6.2 (−39.2, 26.7)	−0.03 (−35.97, 24.42)	0.706
Vegetables (g/d)	94.0 (79.4, 108.5)	97.5 (81.5, 113.5)	0.745	96.5 (81.9, 111.0)	99.9 (82.2, 117.6)	0.762	2.5 (−8.6, 13.6)	2.4 (−9.0, 13.9)	0.03 (−12.38, 19.32)	0.665
White bread (g/d)	6.6 (1.8, 11.3)	7.0 ± (1.4, 12.5)	0.915	6.6 (2.5, 10.8)	6.4 (0.8, 12.0)	0.956	0.0 (−6.1, 6.2)	−0.5 (−8.4, 7.3)	−0.01 (−7.69, 6.56)	0.876
Whole grain bread (g/d)	138.3 (121.9, 154.8)	126.8 (108.0, 145.6)	0.357	122.0 (105.7, 138.2)	123.4 (103.2, 143.6)	0.911	−16.4 (−35.5, 2.8)	−3.4 (−25.1, 18.4)	−0.02 (−27.99, 23.62)	0.867
Low-fat dairy products (g/d)	257.1 (198.7, 307.7)	250.8 (193.9, 307.7)	0.879	268.7 (217.4, 320.1)	306.9 (236.8, 377.1)	0.382	11.7 (−41.2, 64.5)	56.1 (121.1, 36.4)	−0.07 (−108.73, 44.68)	0.410
High-fat dairy products (g/d)	62.5 (34.8, 90.2)	80.6 (38.1, 123.1)	0.477	50.2 (20.5, 79.9)	110.6 (49.0, 172.1)	0.081	−12.3 (−46.8, 22.2)	30.0 (−26.2, 86.1)	−0.13 (−113.09, 12.94)	0.118
Lean meat (g/d)	30.1 (25.7, 34.5)	31.2 (25.6, 36.8)	0.753	29.8 (24.9, 34.8)	33.7 (26.9, 40.5)	0.364	−0.3 (−5.2, 4.7)	2.5 (−2.3, 7.3)	−0.07 (−10.13, 3.85)	0.376
Red/processed meat (g/d)	59.0 (51.0, 67.0)	56.9 (49.6, 64.2)	0.705	54.5 (46.8, 62.2)	54.9 (47.7, 62.0)	0.951	−4.5 (−12.4, 3.5)	−2.1 (−9.7, 5.5)	−0.03 (−11.99, 7.98)	0.691
Sweets (g/d)	26.2 (21.6, 30.9)	19.6 (17.3, 21.9)	0.012	19.8 (17.4, 22.3)	23.8 (19.3, 28.2)	0.127	−6.4 (−11.2, −1.6)	4.1 (−0.6, 8.9)	−0.14 (−8.95, 1.33)	0.145
Salty snacks (g/d)	17.2 (14.2, 20.2)	15.2 (12.4, 18.0)	0.342	13.1 (10.9, 15.4)	16.9 (13.6, 20.3)	0.066	−4.0 (−7.1, −1.0)	1.7 (−2.4, 5.8)	−**0.18 (**−**7.69,** −**0.07)**	**0.046**

Data are presented as mean (95% CI).

* Differences in consumption of food and beverages between the intervention group and the control group, analyzed using independent sample *t*-tests.

# Intervention effect analyzed as difference in mean scores at baseline and after 6 months of follow-up in each variable (of each group) adjusted for baseline scores, age, sex, maternal-, and paternal educational level using linear regression models.

CI: confidence intervals.

*p*-value < 0.05 was considered as statistically significant.

### Change in nutrient intake and BMI z-score

According to results presented in Table 5, the intervention resulted in a significant decrease in total daily energy intake (kJ) in the intervention group compared to the control group (β = –0.17 (95% CI –2,131, –123)). Compared to the control group, the intervention group also significantly decreased the total intake of carbohydrates (β = –0.17 (95% CI –61.97, –6.23)), including mono- and disaccharides (β = –0.21 (95% CI –42.13, –7.29)) and sucrose (β = –0.24 (95% CI –21.26, –4.73)), and the intake of SFAs (β = –0.17 (95% CI –7.95, –0.11)). Finally, results showed an overall decrease in BMI and BMI *z*-score after the 6-month intervention in the intervention group compared to the control group (β = –0.50 (95% CI –1.25, –0.39) and β = –0.21 (95% CI –0.25, –0.10), respectively). See Table 5 for more information about results.

**Table 5:** Changes in body mass index and nutrient intake during the 6-month intervention period

	Baseline			6 months of follow-up			Within-group mean changes after 6 months			
	Intervention group	Control group	*P* *	Intervention group	Control group	*P* *	Intervention group	Control group	β (95% CI)	*P* ^#^
BMI (kg/m^2^)	23.8 (23.0, 24.6)	23.1 (22.4, 23.9)	0.129	24.0 (23.1, 24.8)	24.1 (23.3, 24.8)	0.860	0.2 (−0.1, 0.4)	0.9 (0.6, 1.2)	**−0.50 (−1.25, −0.39)**	**<0.001**
BMI *z*-score	2.04 (1.93, 2.15)	2.02 (1.92, 2.13)	0.825	1.93 (1.82, 2.04)	2.07 (1.97, 2.16)	0.061	−0.11 (−0.16, −0.06)	0.04 (0.00, 0.09)	**−0.21 (−0.25, −0.10)**	**<0.001**
Energy (kJ)	8,096 (7,428, 8,763)	8,159 (7,431, 8,886)	0.899	6,978 (6,424, 7,532)	8,226 (7,164, 9,288)	0.032	−1,118 (−1,575, −662)	67 (−893, 1,027)	**−0.17 (−2131.15, −123.01)**	**0.028**
Total protein (g)	83.3 (76.9, 89.7)	83.7 (76.7, 90.8)	0.919	76.9 (70.6, 83.2)	87.4 (76.7, 98.1)	0.081	−6.4 (−10.7, −2.1)	3.7 (−5.9, 13.2)	−0.14 (−19.99, 0.46)	0.061
Total carbohydrates (g)	243.7 (221.9, 265.5)	247.0 (223.1, 270.8)	0.839	203.5 (186.7, 220.3)	242.1 (211.2, 273.0)	**0.024**	−40.2 (−56.6, −23.8)	−4.9 (−29.6, 19.8)	**−0.17 (−61.97, −6.23)**	**0.017**
Starch (g)	120.7 (110.5, 131.0)	120.9 (109.2, 132.6)	0.977	107.4 (98.5, 116.3)	118.5 (104.3, 132.7)	0.174	−13.3 (−22.5, −4.1)	−2,4 (−14.3, 9.5)	−0.10 (−23.83, 4.65)	0.185
Mono- and disaccharides (g)	118.6 (103.8, 133.5)	121.4 (105.9)	0.796	92.6 (82.8, 102.3)	120.0 (101.1, 138.9)	**0.008**	−26.1 (−38.0, −14.1)	−1,4 (−17.4, 14.6)	**−0.21 (−42.13, −7.29)**	**0.006**
Sucrose (g)	51.1 (43.8, 58.5)	51.7 (44.1, 59.2)	0.917	35.9 (30.8, 41.0)	49.5 (41.3, 57.7)	**0.004**	−15.3 (−22.3, −8.2)	−2,2 (−9.2, 4.8)	**−0.24 (−21.26, −4.73)**	**0.002**
Fiber (g/1,000MJ)	2.9 (2.8, 3.0)	2.9 (2.7, 3.0)	0.627	3.0 (2.8, 3.1)	2.8 ± (2.6, 3.0)	0.141	0.1 (−0.1, 0.2)	−0,1 (−0.2, 0.1)	0.14 (−0.03, 0.41)	0.081
Total fat (g)	63.6 (57.4, 69.8)	63.5 (57.2, 69.8)	0.998	55.3 (50.4, 60.2)	66.0 (56.0, 76.1)	**0.047**	−8.26 (−12.7, −3.8)	2.5 (−8.0, 12.9)	−0.16 (−19.86, 0.66)	0.066
MUFAs (g)	23.3 (20.9, 25.7)	23.1 (20.7, 25.4)	0.897	20.2 (18.3, 22.0)	24.0 (20.3, 27.7)	0.055	−3.1 (−4.8, −1.3)	0.9 (−3.1, 4.9)	−0.15 (−7.25, 0.43)	0.081
SFAs (g)	25.7 (23.2, 28.3)	26.0 (23.4, 28.6)	0.890	22.4 (20.2, 24.5)	27.0 (23.2, 30.8)	**0.029**	−3.4 (−5.3, −1.4)	1.0 (−2.9, 4.9)	**−0.17 (−7.95, −0.11)**	**0.044**
PUFAs (g)	8.3 (7.4, 9.3)	8.2 (7.3, 9.2)	0.896	7.4 (6.6, 8.2)	8.6 (6.7, 10.6)	0.220	−1.0 (−1.6, −0.3)	0.4 (−1.6, 2.4)	−0.11 (−3.19, 0.74)	0.220
*n*−3 fatty acids (g)	0.67 (0.40, 0.93)	0.54 (0.39, 0.69)	0.436	0.69 (0.46, 0.91)	0.64 (0.35, 0.93)	0.774	0.02 (−0.22, 0.26)	0.10 (−0.20, 0.39)	0.02 (−0.30, 0.40)	0.789
*n*−6 fatty acids (g)	1.11 (0.83, 1.40)	0.93 (0.74, 1.12)	0.301	0.98 (0.72, 1.24)	1.06 (0.66, 1.46)	0.729	−0.13 (−0.40, 0.13)	0.13 (−0.31, 0.57)	−0.06 (−0.64, 0.30)	0.478
Cholesterol (mg)	250.4 (222,8, 277.9)	281.7 (234.8, 328.5)	0.236	249.1 (212.6, 285.6)	298.3 (232.8, 363.8)	0.176	−40.2 (−56.6, −23.8)	16.6 (−50.9, 84.2)	−0.08 (−99.95, 36.52)	0.359
E% protein	17.8 (17.2, 18.3)	17.6 (17.0, 18.2)	0.698	18.7 (18.2, 19.2)	18.2 (17.6, 18.9)	0.228	1.0 (0.4, 1.5)	0.6 (0.2, 1.1)	0.06 (−0.43, 0.99)	0.435
E% Fat	28.9 (27.8, 30.0)	28.7 (27.6, 29.9)	0.847	29.3 (28.2, 30.3)	29.4 (28.3, 30.4)	0.900	0.4 (−0.7, 1.5)	0.6 (−0.5, 1.8)	0.00 (−1.36, 1.40)	0.980
E% SFAs	11.6 (11.2, 12.1)	11.8 (11.2, 12.3)	0.727	11.8 (11.8, 11.3)	12.1 (11.5, 12.6)	0.429	0.1 (−0.4, 0.7)	0.3 (−0.3, 0.9)	−0.04 (−0.87, 0.56)	0.662
E% MUFAs	10.6 (10.1, 11.1)	10.4 (9.9, 10.9)	0.669	10.7 (10.2, 11.2)	10.7 (10.2, 11.2)	0.987	0.1 (−0.4, 0.6)	0.3 (−0.2, 0.8)	0.00 (−0.61, 0.64)	0.962
E% PUFAs	3.8 (3.5, 4.0)	3.7 (3.5, 3.9)	0.624	3.9 (3.6, 4.2)	3.7 (3.5, 3.9)	0.276	0.1 (−0.1, 0.3)	0.0 (−0.2, 0.2)	0.06 (−0.16, 0.42)	0.360
E% Carbohydrates	51.0 (49.7, 52.3)	51.4 (50.1, 52.6)	0.706	49.6 (48.5, 50.8)	50.2 (48.9, 51.5)	0.531	−1.4 (−2.5, −0.2)	−1.2 (−2.4, 0.0)	−0.04 (−1.90, 1.07)	0.579
E% Sucrose	10.4 (9.4, 11.3)	10.7 (9.5, 11.8)	0.686	8.6 (7.9, 9.5)	10.1 (9.2, 11.1)	**0.021**	−1.7 (−2.7, −0.7)	−0.5 (−1.5, 0.5)	**−0.19 (−2.55, −0.26)**	**0.017**

Data are presented as mean (95% CI).

BMI: body mass index; E%: percentage of total energy; g: grams: SFAs: saturated fatty acids; MUFAs: monounsaturated fatty acids; PUFAs: polyunsaturated fatty acids; kJ: kilojoule; g: grams; mg: milligrams; MJ: megajoule.

* Significant difference (*P* < 0.05) between the intervention group and the control group, analyzed using independent sample *t*-tests.

# Significant difference (*P* < 0.05) between mean scores at baseline and after 6 months of follow-up in each variable (of each group) adjusted for baseline scores, age, sex, maternal-, and paternal educational level using linear regression models.

*p*-value < 0.05 was considered as statistically significant.

### Identification of intervention effects using multiple-group SEM of latent means

Pathways analyses, presented in Table 6, show that the intervention directly predicted BMI *z*-score (β = –0.17), total carbohydrates (β = –0.20), mono- and disaccharides (β = –0.26), and sugar (β = –0.22) after intervention. In addition, post-treatment levels of energy (β = –0.65), SFAs (β = 0.43), and total carbohydrates (β = 0.41) were directly associated with BMI *z*-score after intervention. Age and sex were directly linked to energy, mono- and disaccharides, and baseline BMI *z*-score. Age and sex indirectly predicted BMI *z*-score after intervention via energy, SFAs, and baseline BMI *z*-score (Table 6).

**Table 6:** Direct and indirect effects of the parsimonious structural equation model

Pathways	β	SE	*P*
Direct effects			
Intervention → Total carbohydrates	−0.20	0.09	0.026
Intervention → Mono- and disaccharides	−0.26	0.08	0.002
Intervention → Sucrose	−0.22	0.09	0.011
Intervention → BMI *z*-score post-intervention	−0.17	0.04	<0.001
Age → Energy	−0.23	0.09	0.007
Age → SFAs	−0.20	0.09	0.024
Age → Mono- and disaccharides	−0.13	0.06	0.040
Age → BMI *z*-score baseline	−0.36	0.08	<0.001
Sex → Energy	0.22	0.09	0.011
Sex → SFAs	0.18	0.08	0.008
Sex → Mono- and disaccharides	0.19	0.09	0.023
Sex → BMI *z*-score baseline	0.34	0.07	<0.001
Energy → BMI *z*-score post-intervention	−0.65	0.24	0.007
SFAs→ BMI *z*-score post-intervention	0.43	0.13	0.001
Total carbohydrates → BMI *z*-score post-intervention	0.41	0.19	0.032
BMI baseline → BMI *z*-score post-intervention	0.85	0.03	<0.001
Indirect effects			
Age → BMI *z*-score post-intervention	−0.55	0.02	<0.001
Age → Energy → BMI *z*-score post-intervention			
Age → SFAs → BMI *z*-score post-intervention			
Age → BMI *z*-score baseline → BMI *z*-score post-intervention			
Sex → BMI *z*-score post-intervention	−0.22	0.06	<0.001
Sex → Energy → BMI *z*-score post-intervention			
Sex → SFAs → BMI *z*-score post-intervention			
Sex → BMI baseline → BMI *z*-score post-intervention			

BMI: body mass index; SFAs: saturated fatty acids; SE: standard error.

## Discussion

The present study assessed the effects of a complex intervention program on meal pattern, food and beverage availability and consumption, nutrient intake, and weight status among children with overweight/obesity. Overall, the intervention successfully increased frequency of evening meals, decreased availability and consumption of salty snacks, and increased consumption of water in the intervention group compared to the control group after the intervention period. The main finding of the present study, which seemed to explain the observed decrease in BMI *z*-score during the intervention period, was a decrease in total energy intake, total carbohydrate intake, intake of mono- and disaccharides and sucrose, and intake of SFAs during the intervention period among participants in the intervention group compared to the control group.

Baseline results from the intervention and control group highlight the potential of reducing energy intake to improve weight status, as both groups confirmed a higher mean intake of total energy intake, total protein and carbohydrate intake, total intake of fat, intake of SFAs and cholesterol, and a similar intake of added sugar compared to estimates from the national dietary survey among 9-year-old Norwegian children (43).

The reduction in BMI *z*-score reported in the present study is in line with the effect size reported by other intervention studies focusing on behavior change among overweight and obese 6–11-year-olds, but results from previous studies reveal large variations in mean effects (from 0.1 to –0.71) (27). The reasons for the variations in effect could be due to differences between the intervention elements, age of participants, and setting. Further research has been warranted to identify optimal methods and strategies for weight management among children with overweight/obesity, including degree of parental involvement and how health practitioners can work with parents as agents of change in order to support health behaviors in their children (27, 30).

Another recent systematic review on family-based nutrition interventions for obesity prevention concluded that most successful intervention studies included a cluster of behavior change techniques, such as setting family-based goals, modifying home food environment, hands-on approaches to teaching nutrition (games and group-based activities), and fruit and vegetable vouchers (44). Using active family-based learning involving meal preparation and cooking has been associated with increased parent–child communication, teamwork, and inspiration to implement healthy eating as part of everyday life (45, 46), which might explain positive changes in nutrient intake observed in the present study. This conclusion is also supported by results from a recent systematic review, which presented experimental learning as a useful strategy to improve knowledge, attitudes, and behaviors toward healthy eating among children (47).

Unfortunately, due to the design of our study, we were unable to entangle the effects of parental involvement on outcomes in our study. However, in line with the program components used in our study, previous weight management studies among children with overweight/obesity have emphasized the importance of involving parent as the agent of change through nutrition education sessions, using an authoritative parenting style, enhancing positive parenting skills and child behavior management strategies (30, 48). Furthermore, a 12-month intervention study showed that allocating children with overweight/obesity to multiple-family intervention significantly decreased waist circumference, but not BMI, then those allocated into single-family interventions (49). However, no additional long-term effect of group vs individual family intervention in treatment of childhood obesity was shown at 36 months follow-up (50).

A systematic review of primary healthcare obesity interventions also indicated a positive relationship between hours of contact and treatment effect (51). This conclusion has been supported by previous findings of a study based on primary care screening for identification and follow-up by brief parent counseling (four consultations over a 12-week period) of children, aged 5–10 years old, with overweight and obesity, which did not result in improved BMI, physical activity, or nutrition among participants (52). Thus, the high frequency of individual consultations and group-based courses combined may most likely have contributed the positive treatment effects observed in the present study. Results from our study in combination with previously reported findings may therefore indicate that current policies in several countries that ensure use of national screening programs in combination with brief, individualized prevention measures by primary care sector to reduce childhood obesity should be reevaluated.

Our findings also identified that age and sex to be relevant determinants of nutrient intake and weight status once higher age and being female predicted lower intake macronutrients during the intervention period and consequently reduced BMI *z*-score. Relatively few studies have previously investigated sex-based differences in childhood obesity, but the importance of including sex-specific focus to increase effectiveness of future family-based interventions has been warranted (53). Previous studies have also shown that specific approaches may be important for some age groups, but not for others, depending on their level of self-regulation skills: i.e. setting family-based goal and including hands-on nutrition activities such as games seem to effectively contribute to change dietary habits among younger children, but not adolescents (54, 55). Findings from previously published studies among children and adolescents suggest that parental support as an influence on child health behaviors tends to peak at age 12 (56), which support the use of a family-based approach in the present study.

### Strength and limitations of study

A limitation of the current controlled trial is the lack of a randomized design, and a time lag between data collection and publication. A recent systematic review has also confirmed that relatively few randomized controlled trials testing the efficacy of family-based nutrition interventions targeting school aged children with overweight/obesity have been published (44). On the other hand, the study design used in the present study was tailored and adapted for intervention in ordinary community-based settings, which may increase the likelihood of implementation in regular services. Another limitation of the study was that despite mandatory screening of height and weight among Norwegian preschoolers and 3rd graders, the recruitment process in regular services in several different municipalities did not allow us to control this process and ensure that all children and families in the target group had been invited to participate in the present study. Moreover, high drop-out rates and low number of participants in each group who completed the study are other limitations that should be mentioned. However, the inclusion of 143 participants yielded an adequate power of 94%. In line with our study, most previously published results from child obesity prevention trials are limited by low sample size (57). Moreover, as municipal healthcare institutions have a responsibility to prevent and treat overweight and obesity among all age groups (22), we used a waiting list design, which did not allow long-term controlled follow-up.

A strength of the present study was the use of a structural theory and evidence framework for studying development, implementation, and evaluation. Moreover, results from the pilot study provided information on opportunities and barriers to tailor the intervention to the local context in Norwegian municipalities and to adapt the intervention tools and materials to health personnel conducting the study and to the target group (31). Another strength was the use of objectively measured height and weight by trained public health nurses, and the use of a validated and comprehensive FFQ, which provided estimates of nutrient intake (37). Moreover, the use of structural equation modeling as a statistical approach for the evaluation of multiple relationships between variables provided increased knowledge about pathways of change.

### Future perspectives

Considering that overweight and obesity among children is a global problem and to limit stains on the healthcare system, future interventions should reevaluate strategies used to conduct family-based treatment programs targeting children with overweight/obesity. Emerging evidence indicates that the incorporation of digital technology together with conventional treatments may be an acceptable approach to increase effectiveness in weight management intervention studies (58). In addition, there is a need to evaluate possible long-term effects and how interventions effects might vary among different sub-groups, such as non-traditional families, and racial and ethnic minorities.

## Conclusion

A positive intervention effect was observed on evening meal pattern, availability and consumption of salty snacks, and consumption of water. Moreover, estimates indicating a decreased total intake of energy and carbohydrates, including total carbohydrate intake, intake of mono- and disaccharides and sucrose, and intake of SFAs seem to explain the observed decrease in BMI *z*-score during the intervention period. Thus, the creation of environments supportive of healthier behaviors seems to mitigate challenges related to childhood overweight and obesity.
